# The Anti-Inflammatory Effect of Octyl Gallate Through Inhibition of Nuclear Factor-κB (NF-κB) Pathway in Rat Endometriosis Model

**Published:** 2020

**Authors:** Arleni Bustami, Wahyu Pangestuti Lestari, Cicilia Febriani Hayuningrum, Puspita Eka Wuyung, Heri Wibowo, R. Muharam Natadisastra

**Affiliations:** 1- Integrated Laboratory of Medical Faculty, Universitas Indonesia, Jakarta, Indonesia; 2- Master Program in Biomedical Sciences, Faculty of Medicine, Universitas Indonesia, Jakarta, Indonesia; 3- Departement of Pathology Anatomy, Faculty of Medicine, Universitas Indonesia, Jakarta, Indonesia; 4- Departement of Parasitology, Faculty of Medicine, Universitas Indonesia, Jakarta, Indonesia; 5- Departement of Obstetrics and Gynecology, Faculty of Medicine, Universitas Indonesia, Cipto Mangunkusumo National General Hospital, Jakarta, Indonesia

**Keywords:** Chronic inflammation, COX-2, Endometriosis, NF-κB, Octyl gallate

## Abstract

**Background:**

Endometriosis is a chronic inflammatory condition associated with an increased risk of epithelial ovarian cancer. Our previous studies found that the anti-inflammatory effect of octyl gallate in endometriosis cell culture was more effective than gallic acid and heptyl gallate. This study aimed to analyze the anti-inflammatory effect of octyl gallate in rat endometriosis model.

**Methods:**

Thirty female Wistar rats were randomly divided into three groups. Group I was the sham-operated group, group II was the surgically-induced endometriosis group, whereas group III was the surgically-induced endometriosis group and each rat was administered with 20 *mg* of octyl gallate dissolved in 1 *ml* Na-CMC via oral gavage once a day for 30 days. When all rats were euthanized, the endometrial tissue from group I and last two groups were collected for further analysis. TNF-α levels were measured using Luminex, while non-phosphorylated NF-κB and COX-2 levels were analyzed using ELISA.

**Results:**

The average of non-phosphorylated NF-κB levels in group III (4.970±0.971 *pg/mgP*) was significantly higher than group II (3.97±0.656 *pg/mgP*). Moreover, the proportion of rats with the high level of non-phosphorylated NF-κB in group III was 45.6% higher than group II (p<0.05). The proportion of rats with the high level of COX-2 in group III was 22.3% lower than group II (p<0.05). However, there was no significant difference in TNF-α levels between all groups.

**Conclusion:**

The anti-inflammatory effect of octyl gallate may has effects in NF-κB activation and reduction of COX-2 levels in rat endometriosis model.

## Introduction

Endometriosis is a gynecologic condition characterized by the formation of endometrial like tissue outside the uterine cavity. Endometriosis affects 5–10% of all women of reproductive age and causes several symptoms including chronic pelvic pain, dysmenorrhea, and dyspareunia. Endometriosis is associated with infertility and an increased risk of epithelial ovarian cancer (EOC) (1, 2).

Chronic inflammation in the peritoneal cavity has an important role in endometriosis progression. Inflammatory mediators, such as IL-1β and TNF-α, will activate transcription factor NF-κB that creates a positive feedback loop to increase various inflammatory mediators, including TNF-α, IL-1, IL-6, and IL-12 (3, 4). Inflammatory condition can cause the formation of endometrial cell adhesion sites due to tissue damage process. Moreover, the increase of NF-κB activation is associated with the upregulation of COX-2 levels, which plays an important role in Prostaglandin E_2_ (PGE_2_) synthesis (1, 5, 6). The upregulation of PGE_2_ levels can enhance estrogen hormone and Vascular endothelial growth factor (VEGF) induced-angiogenesis, along with apoptosis and lymphocyte proliferative inhibition (5). This NF-κB induced-chronic inflammation then promotes the development of endometrial cysts and causes more profound clinical signs.

The current treatment of endometriosis, including hypoestrogenic induction and analgesic administration, is known to be ineffective because the treatment is unable to remove existing endometriosis tissue and provides adverse effects on long-term administration. Meanwhile, the removal of endometriosis tissue through surgery is also not optimal because the recurrence rate after therapy still reaches 40–80% (1). This underlies the need to develop the other potential alternative treatment.

Octyl gallate is one of the gallic acid derivates, widely contained in natural sources, such as green tea, grapes, pineapple, and strawberries. Gallic acid is known to have an antioxidant, anti-inflammatory, and proapoptotic effect in cancer cells (7). Our previous studies on endometriosis primary cells culture have proven octyl gallate was more effective compared to gallic acid and heptyl gallate in suppressing inflammatory regulation, particularly in the expression of NF-κB, IL-6, COX-2, mRNA and a noticeable decrease in endometriosis cell viability was observed as tested by 3-(4,5-di-methylthiazol-2-yl)-2,5-diphenyltetrazolium bromide (MTT) assay (8–10). In addition, octyl gallate was also shown to have a more stable affinity and stronger bond to inhibit NF-κB compared to the other compounds (9). However, *in vivo* experiments on the anti-inflammatory effect of octyl gallate in endometriosis have never been studied before.

This study aimed to analyze the anti-inflammatory effect of octyl gallate through the inhibitions of the NF-κB pathway in rat endometriosis model.

## Methods

### Materials:

Octyl gallate 99% and Sodium carboxy methyl cellulose (Na-CMC) were purchased from Sigma-Aldrich (Singapore); Nuclear factor-kappa B (NF-kB) ELISA kit was purchased from My Biosource (MBS722386, USA) with range of assay: 0–1000 *ng/ml*; COX-2 ELISA kit was purchased from My BioSource (MBS725633, USA) with range of assay: 0–100 *ng/ml*; TNF-α Magnetic Luminex Multiplex Assay kit was purchased from R&D Systems (LXSARM-03, USA).

### Experimental design:

Thirty female Wistar rats, aged 7–8 weeks with an average weight of 150–200 *g*, were obtained from the Institute for Health Research and Development, Indonesia. All rats were housed with free access to food and water. The temperature was maintained at room temperature (23–25°*C*) in a simulated daylight condition (12 *hr* light: 12 *hr* dark). All protocols in this study were approved by the Health Research Ethics Committee of the Faculty of Medicine, Universitas Indonesia, with ethical code 0661/UN2.F1/ETIK/2018.

A week after the acclimatization, all rats were randomly divided into three groups, defined as the followings:

Group I: Underwent laparotomy (Sham-operated group)
Group II: Surgically-induced endometriosis administered with Na-CMC
Group III: Surgically-induced endometriosis administered with octyl gallate dissolved in Na-CMC

### Induction of endometriosis:

**Figure 1.**
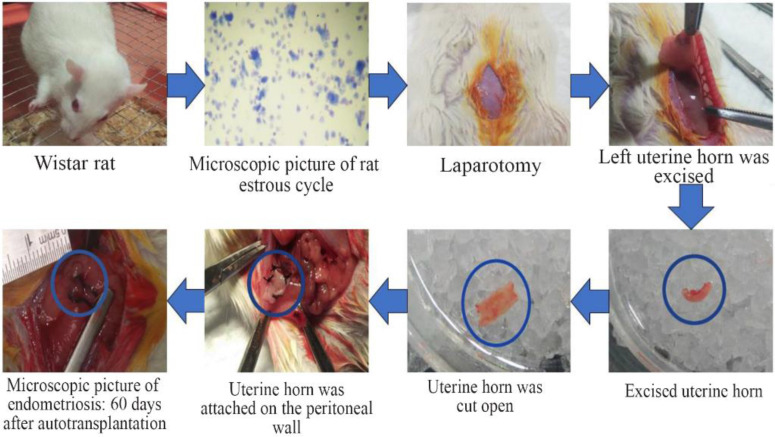
Surgically-induced endometriosis (Autotransplantation). The autotransplantation was performed in rats during their estrous cycle, proven by a vaginal pap smear. Rats were anesthetized with ketamine at dose 73 *mg/Kg* BW and xylazine at dose 8.8 *mg/Kg* BW, then underwent a laparotomy. The left uterine horn was excised±0.5 *mm* long, placed in a petri dish containing 0.9% NaCl, then cut open. The cut-opened horn was attached on the right peritoneal wall near a vein using a 3-0 size silk thread with the endometrial tissue facing the peritoneal wall. The formation of the endometrial cyst was evaluated with second laparotomy 60 days after autotransplantation

**Figure 2.**
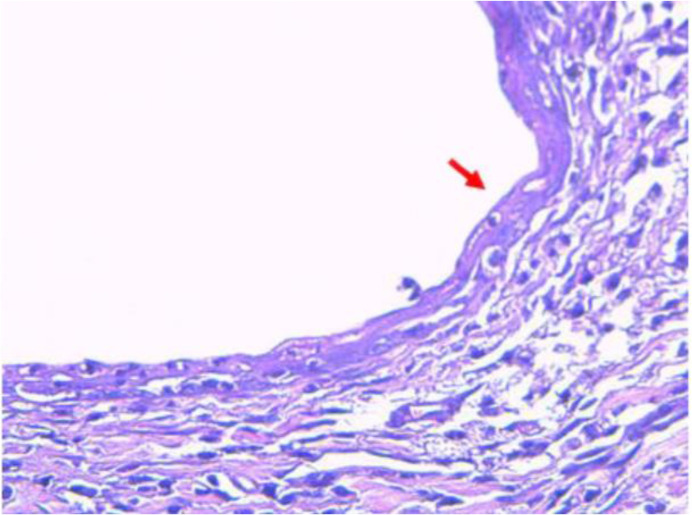
Hematoxylin-eosin (HE) staining of rat surgically-induced endometriosis (40× magnification). HE staining from our previous study proved that endometrial cyst induced with autotransplantation was truly an endometriosis tissue. The red arrow sign indicates the presence of the endometrial gland

Group II and III underwent autotransplantation technique to develop endometriosis tissue (Figure 1). The autotransplantation process was performed in rats during their estrous cycle, proven by a vaginal pap smear. Surgically-induced endometriosis method by autotransplantation was adopted from Fischer et al. (10) with minor modification. Rats were anesthetized with ketamine at dose 73 *mg/Kg* BW and xylazine at dose 8.8 *mg/Kg* BW. The left uterine horn was excised±0.5 *mm* length, placed in a petri dish containing 0.9% NaCl, then cut open. The cut-opened horn was attached on the right peritoneal wall near a vein using a 3-0 size silk thread with the endometrial tissue facing the peritoneal wall. On the other hand, group I underwent laparotomy without the autotransplantation of uterine tissue. Injection of antibiotics 100 *mg/Kg* BW was given intraperitoneally (IP) before the abdominal wall was closed. The abdominal wall was then sutured using 3-0 size chromic catgut thread and the outer skin was sutured using 3-0 size silk thread. For the next two days, rats were injected with antibiotics and evaluated for any sign of illness. Two months after the endometriosis induction, the second laparotomy was performed in groups II and III to evaluate the formation of the endometrial cyst. The endometriosis lesions were previously confirmed through HE tissue staining (Figure 2).

### Octyl gallate induction:

Group II was administered with 1 *ml* of Na-CMC solution, while group III was administered with 20 *mg* of octyl gallate dissolved in 1 *ml* Na-CMC via oral gavage once a day for 30 days.

### Tissue collection:

All rats were sacrificed using high-dose ketamine. The endometrial tissues of the group I, along with endometriosis tissue of the group II and III were collected. Then, tissues were washed in 0.9% NaCl and immediately stored in PBS with a ratio of 100 *mg/ml*. Sample tissues were homogenized using a high-pressure sonication method, then centrifuged at 5000 *rpm* for 10 *min* at 4°*C*. The supernatant was separated and analyzed.

### Inflammatory mediator measurement:

Inflammatory mediators NF-κB and COX-2 were analyzed using ELISA kits. Pro-inflammatory mediators, TNF-α, were analyzed using Magnetic Luminex Multiplex Assay kit. Inflammatory mediator levels were divided by total protein concentration of each sample. Total protein levels were measured with spectrophotometry at 280 *nm* wavelength using the Warburg-Christian method.

### Statistical analysis:

Statistical analysis was performed using Statistical Package for the Social Sciences (SPSS) software version 23. The mean difference of non-phosphorylated NF-κB, COX-2, and TNF-α levels among three groups was analyzed using Analysis of Variance (ANOVA), while the different proportions of the low and high level of non-phosphorylated NF-κB levels (Cut off point=5.002 *pg/mgP*), COX-2 levels (Cut off point=16.151 *ng/mgP*), and TNF-α levels (Cut off point=75.888 *pg/mgP*) were analyzed using Fisher’s Exact Test. A value of p<0.05 was considered statistically significant.

## Results

Rat in group I of this study underwent laparotomy as a sham group, while group II and III underwent surgically-induced endometriosis using autotransplantation technique (Figure 1). This surgically-induced endometriosis method was proved to be able to develop endometriosis lesions, confirmed through HE tissue staining from our previous study (Figure 2).

**Figure 3.**
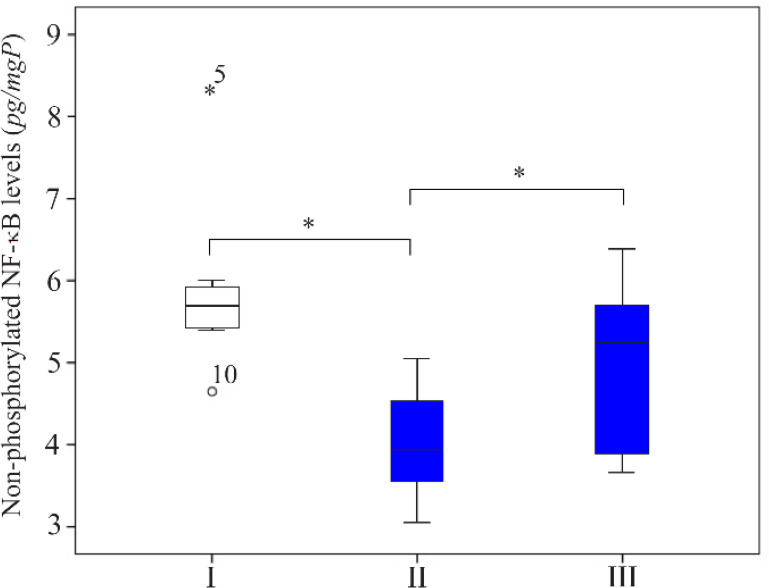
The comparison of non-phosphorylated NF-κB levels in the supernatant of homogenized rat endometriosis tissue. I: sham-operated group; II: Surgically-induced endometriosis; III: Surgically-induced endometriosis+octyl gallate (20 *mg* octyl gallate dissolved in 1 *ml* Na-CMC, administered via oral gavage once a day for 30 days). The average (Mean±standard deviation) of non-phosphorylated NF-κB levels in group I (5.827±0.909 *pg/mgP*), group II (3.97±0.656 *pg/mgP*), and group III (4.970±0.971 *pg/mgP*) (*p<0.05)

The average of non-phosphorylated NF-κB levels in group I (5.827±0.909 *pg/mgP*) was significantly higher compared to group II (3.908± 0.664 *pg/mgP*) (p<0.05), indicated the increase of NF-κB activation in rat endometriosis model. Oral octyl gallate induction significantly upregulated the non-phosphorylated NF-κB levels in group III (4.970±0.971 *pg/mgP*) compared to group II (p< 0.05), indicated the decrease of NF-κB activation (Figure 3).

**Figure 4.**
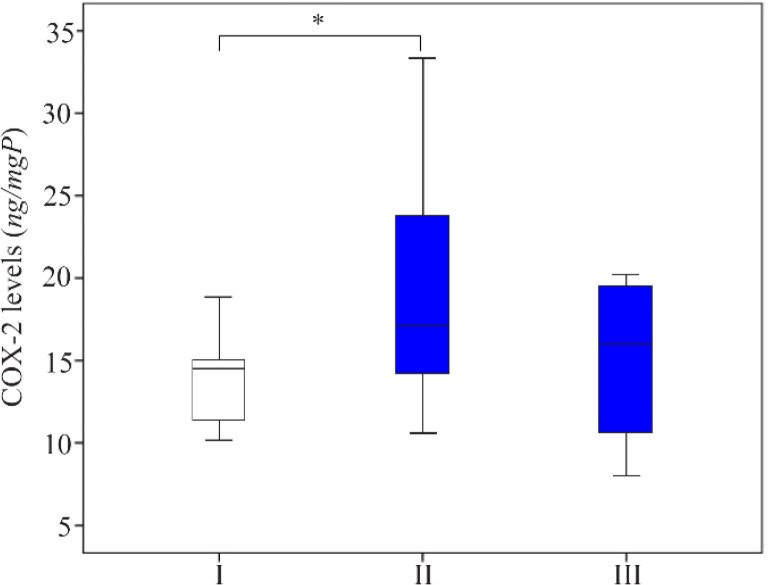
The comparison of COX-2 levels in the supernatant of endometriosis tissue of homogenized rat. I: sham-operated group; II: Surgically-induced endometriosis; III: Surgically-induced endometriosis+octyl gallate (20 *mg* octyl gallate dissolved in 1 *ml* Na-CMC, administered via oral gavage once a day for 30 days). The average (Mean±standard deviation) of COX-2 levels in group I (13.897±2.621 *ng/mgP*), group II (19.285±7.575 *ng/mgP*), and group III (14.721±4.940 *ng/mgP*) (*p<0.05)

**Figure 5.**
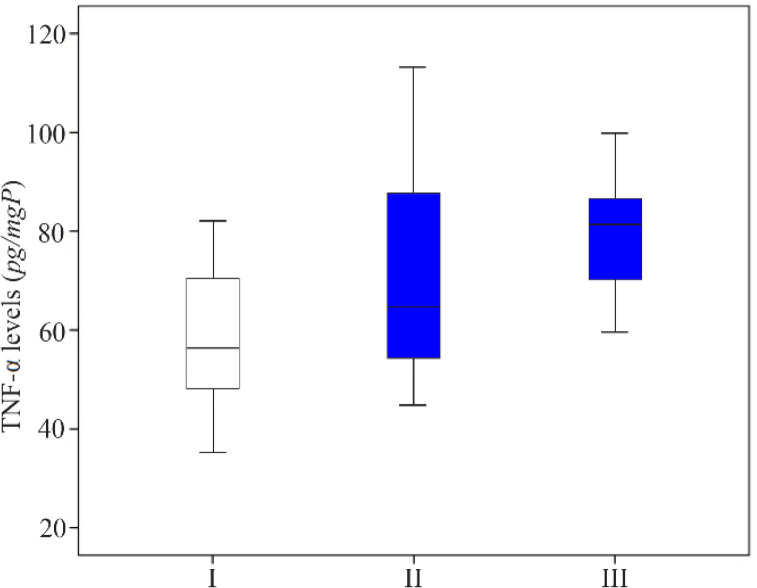
The comparison of TNF-α levels in the supernatant of endometriosis tissue in homogenized rat. I: sham-operated group; II: Surgically-induced endometriosis; III: Surgically-induced endometriosis+octyl gallate (20 *mg* octyl gallate dissolved in 1 *ml* Na-CMC, administered via oral gavage once a day for 30 days). The average (Mean±standard deviation) of TNF-α levels in group I (58.909±14.188 *pg/mgP*), group II (73.564±23.936 *pg/mgP*), and group III (79.439±12.881 *pg/mgP*)

The decrease of non-phosphorylated NF-κB levels in group II was followed by a significant increase of COX-2 levels (19.285±7.575 *ng/mgP*) compared to group I (13.897±2.621 *ng/mgP*). Oral octyl gallate induction then reduced the average of COX-2 level in group III (14.721±4.940 *ng/mgP*) in comparison to group II (Figure 4). Furthermore, the average of TNF-α levels was elevated in group II (73.564±23.936 *pg/mgP*) comparing to group I (58.909±14.188 *pg/mgP*), but the average levels among three groups were not significantly different (Figure 5).

**Figure 6.**
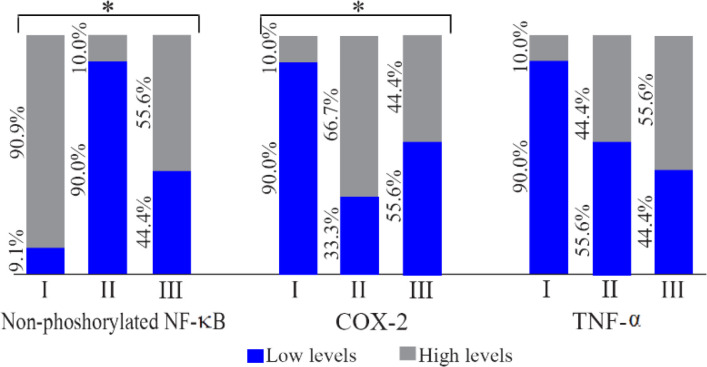
The comparison of the different proportions of the high and low levels of non-phosphorylated NF-κB, COX-2, and TNF-α in the supernatant of endometriosis tissue of homogenized rat. I: sham-operated group; II: Surgically-induced endometriosis; III: Surgically-induced endometriosis+octyl gallate (20 *mg* octyl gallate dissolved in 1 *ml* Na-CMC, administered via oral gavage once a day for 30 days). The low category levels were ≤cut off point, while the high category levels were >cut off point. The cut off point of non-phosphorylated NF-κB was 5.002 *pg/mgP*, COX-2 was 16.151 *ng/mgP*, and TNF-α was 75.888 *pg/mgP* (*p<0.05)

The different proportions of non-phosphorylated NF-κB, COX-2, and TNF-α levels between the three groups were used for further analysis. Figure 6 shows that the proportion of rats with high level of non-phosphorylated NF-ĸB in group II was 80.9% lower than those in group I. On the other hand, the proportion of rats with high level of COX-2 was 56.7% higher. Oral octyl gallate intake caused the proportion of rats with the high level of NF-κB in group III to be 45.6% higher than group II, which led to the reduction of the proportion of rats with the high level of COX-2 in group III (44.4%) compared to group II (66.7%). However, there were no significant differences in TNF-α high category levels between the three groups.

## Discussion

The NF-ĸB signaling pathway is the main transcription factor that plays a major role in many chronic inflammatory diseases, such as endometriosis (13). The enhancement of NF-ĸB activation in endometriosis leads to the maintenance and progression of endometriosis lesions, making NF-ĸB a potential drug target in endometriosis condition. The NF-ĸB proteins are normally sequestered in the cytoplasm with its inhibitory proteins called IĸB family members.

Pro-inflammatory cytokines such as TNF-α, IL-1β, IL-6, and IL-8 could induce the degradation of IĸB, resulting in the nuclear translocation of NF-ĸB members (9, 13). Furthermore, binding of the members of NF-ĸB to DNA promoter would induce the expression of pro-inflammatory cytokines, intercellular adhesion molecules, and angiogenesis factors (11–13). Moreover, high activation of NF-ĸB in endometriosis then increases the synthesis of COX-2 (1, 2, 5, 14, 15). These theories are consistent with our result. In this study, the proportion of rats with the high level of TNF-α and COX-2 was increased in the surgically-induced endometriosis rats compared to the sham-operated rats. In addition, the proportion of rats with the high level of non-phosphorylated NF-ĸB was decreased, indicating that activation of NF-ĸB was elevated in endometriosis rat model.

The previous studies suggested that gallic acid and its derivative were able to inhibit the activation of NF-ĸB (7, 16, 17). This is consistent with our result indicating that octyl gallate was able to inhibit NF-ĸB translocation to the nucleus, illustrated through the significant increase of non-phosphorylated NF-ĸB levels in the rats which were administered with octyl gallate compared to the surgically-induced endometriosis rats. Moreover, our previous study proved that octyl gallate had strong binding power and stable affinity with NF-ĸB, analyzed using in silico docking method. ^9^ This binding inhibited the nuclear translocation of NF-ĸB and suppressed the transcription of pro-inflammatory genes.

Several studies by Wei et al. (18), Kim et al. (19), and Jiang et al. (20) proved that gallic acid induction was able to inhibit inflammatory reaction in rat model that exists in suppressing the inflammatory cytokines, including TNF-α levels. In addition, research by Mori et al. (21) also showed the effectiveness of octyl gallate combined with ferulic acid in reducing TNF-α levels in Alzheimer's mice model. Nevertheless, octyl gallate in this study did not appear to reduce TNF-α levels as it was evident by the average of TNF-α levels in the rats administered with octyl gallate which increased compared to the surgically-induced endometriosis rats. However, the increase was not statistically significant, so octyl gallate likely had no effect on TNF-α levels. TNF-α can act both as one of the activators and the result of the nuclear translocation of NF-κB. Therefore, the ineffectiveness of octyl gallate in suppressing TNF-α levels might happen due to the ability of octyl gallate in inhibiting the activation of NF-κB directly without affecting TNF-α level as its activator.

Despite its ineffectiveness in suppressing TNF-α levels, oral octyl gallate induction was able to reduce COX-2 levels, another inflammation mediator that plays an important role in the progression of endometriosis. This result was consistent with our previous study that showed the effectiveness of octyl gallate in reducing COX-2 levels in endometriosis primary cell cultures (9). In addition, a study by Das et al. (7) also demonstrated that octyl gallate was capable in reducing PGE_2_ levels through the suppression of COX-2 synthesis. COX-2 is one of the enzymes that plays an important role in PGE_2_ synthesis, so the decrease of COX-2 levels in endometriosis tissue can suppress PGE_2_ synthesis. PGE_2_ levels in patients with endometriosis are known to be quite high and trigger immune dysfunction. Consequently, immune cells are not able to eliminate endometrial cysts. Furthermore, the increase of PGE_2_ can promote the development of endometrial cysts through apoptosis inhibition by increasing the anti-apoptotic protein Bcl2 and reducing the pro-apoptotic protein Bax (1, 5, 6). Based on this description, the decreased COX-2 levels in endometriosis induced by octyl gallate can ultimately play an important role in inhibiting the development of endometrial cysts.

The anti-inflammatory effect of gallic acid and its derivatives in rat model was able to decrease the expression of pro-inflammatory cytokines, including TNF-α, which led to the inhibition of phosphorylated NF-ĸB (18). However, our finding suggested that the anti-inflammatory mechanism of octyl gallate in rat endometriosis model involved the direct inhibition of NF-ĸB activation which led to suppression of COX-2 levels.

## Conclusion

Oral administration of octyl gallate was able to reduce COX-2 levels of rat endometriosis tissues through the inhibition of NF-kB activation. However, this study showed that octyl gallate was ineffective in suppressing TNF-α levels. It might be related to the ability of octyl gallate that directly inhibits NF-κB without affecting TNF-α level. However, further studies are needed to prove this mechanism.
